# A systematic review and meta-analysis of the serum lipid profile in prediction of diabetic neuropathy

**DOI:** 10.1038/s41598-020-79276-0

**Published:** 2021-01-12

**Authors:** Zixin Cai, Yan Yang, Jingjing Zhang

**Affiliations:** National Clinical Research Center for Metabolic Diseases, Metabolic Syndrome Research Center, Key Laboratory of Diabetes Immunology, Ministry of Education, and Department of Metabolism and Endocrinology, The Second Xiangya Hospital of Central South University, Hunan, 410011 China

**Keywords:** Endocrinology, Risk factors

## Abstract

Whether the lipid profile in diabetic patients is associated with diabetic neuropathy (DN) development remains ambiguous, as does the predictive value of serum lipid levels in the risk of DN. Here, we performed the first meta-analysis designed to investigate the relationship between DN and the serum levels of triglyceride (TG), total cholesterol (TC), high-density lipoprotein cholesterol (HDL), and low-density lipoprotein cholesterol (LDL). Candidate studies were comprehensively identified by searching PubMed, Embase, Cochrane Library and Web of Science databases up to May 2020. Observational methodological meta-analysis was conducted to assess the relationships of TG, TC, HDL, and LDL levels with DN. Changes in blood lipids were used to estimate the effect size. The results were pooled using a random-effects or fixed-effects model. Potential sources of heterogeneity were explored by subgroup analysis. Various outcomes were included, and statistical analyses were performed using STATA (Version 12.0). Mean differences (MDs) and odds ratios (ORs) with 95% confidence intervals (CIs) were estimated. The Newcastle–Ottawa Scale (NOS) was applied to assess the methodological quality. *I2* statistics were calculated to evaluate statistical heterogeneity. Funnel plots were utilized to test for publication bias. A sensitivity analysis was performed by omitting each study one by one. Thirty-nine clinical trials containing 32,668 patients were included in the meta-analysis. The results demonstrated that DN patients showed higher TG and lower HDL levels (MD = 0.34, 95% CI: 0.20–0.48 for TG; MD = -0.05, 95% CI: -0.08–-0.02, *I*^*2*^ = 81.3% for HDL) than controls. Subgroup analysis showed that patients with type 1 diabetes mellitus (T1DM) neuropathy had elevated TG levels in their serum (MD = 0.25, 95% CI: 0.16–0.35,*I*^*2*^ = 64.4% for T1DM). However, only patients with T1DM neuropathy had reduced serum HDL levels, and there was no significant difference in serum HDL levels between patients with T2DM neuropathy and controls (MD = -0.07, 95% CI: -0.10–-0.03, *I*^*2*^ = 12.4% for T1DM; MD = -0.02, 95% CI: -0.07–0.03, *I*^*2*^ = 80.2% for T2DM). TC and LDL levels were not significantly different between DN patients and controls (MD = -0.03, 95% CI: -0.14–0.09, *I*^*2*^ = 82.9% for TC; MD = -0.00, 95% CI: -0.08–0.08, *I*^*2*^ = 78.9% for LDL). In addition, compared with mild or painless DN patients, those with moderate or severe pain DN pain had significantly reduced serum TC and LDL levels (MD = -0.31, 95% CI: -0.49–-0.13, *I*^*2*^ = 0% for TC; MD = -0.19, 95% CI: -0.32–-0.08, *I*^*2*^ = 0% for LDL). TG levels and HDL levels did not vary considerably between patients with mild or painless DN and those with moderate or severe DN pain patients (MD = 0.12, 95% CI: -0.28–0.51, *I*^*2*^ = 83.2% for TG; MD = -0.07, 95% CI:-0.14–0.01, *I*^*2*^ = 58.8% for HDL). Furthermore, people with higher TG and LDL levels had higher risk of DN (OR = 1.36, 95% CI: 1.20–1.54, *I*^*2*^ = 86.1% for TG and OR = 1.10, 95% CI: 1.02–1.19, *I*^*2*^ = 17.8% for LDL). Conversely, high serum HDL levels reduced the risk of DN (OR = 0.85, 95% CI: 0.75–0.96, *I*^*2*^ = 72.6%), while TC levels made no significant difference with the risk of DN (OR = 1.02, 95% CI: 1.00–1.04, *I*^*2*^ = 84.7%). This meta-analysis indicated that serum lipid profile changes are among the biological characteristics of DN. Lipid levels should be explored as routine laboratory markers for predicting the risk of DN, as they will help clinicians choose appropriate therapies, and thus optimize the use of available resources.

## Introduction

Diabetic neuropathy (DN) is a highly common but often neglected complication of both type 1 diabetes mellitus (T1DM) and type 2 diabetes mellitus (T2DM), affecting an estimated 50% of individuals with diabetes^1^. Its prevalence is more than 2% in the general population^2,3^ and approximately 15% among people over 40 years old^4^. Importantly, patients with peripheral and autonomic neuropathy have a more than twofold increase in their risk of death^5^. DN is a progressive and debilitating disease that can seriously reduce a patient's quality of life and is a key cause of non-trauma related amputations of the lower limbs^6,7^, which means that DN is recognized as the leading contributor to disability in people with diabetes^8^. In addition, one-third of subjects with DN report burning, tingling, shooting or lancing sensations as a symptom and need help alleviating this symptom^9,10^. Painful diabetic neuropathy (PDN) can seriously negatively affect patients’ psychological and physical health, leading to anxiety, depression and sleep disorders^11,12^. The treatment of PDN can be a major challenge for both the clinician and the patient, as such pain is unresponsive or only partially responsive to existing management approaches^13,14^. Owing to the pressing need for a solution to DN, many clinical studies have been carried out to prevent or cure this complication. To date, there is no treatment to prevent the onset of DN other than intensive glycaemic control, which substantially reduces the incidence of DN only in T1DM patients and is minimally effective in preventing DN among patients with T2DM^15–17^. The cause of DN is more complex than dysregulated glucose levels alone. Even patients with good glycaemic control (HbA1c < 5.4%) can still develop DN, suggesting that components other than glycaemic control may be involved in the onset and progression of DN^18^. Recently, some studies have implicated cardiovascular risk factors, such as obesity^19^ and triglycerides (TG)^20^, in the pathogenesis of DN. Therefore, it is essential to understand whether lipid levels modulate DN progression.

Changes in serum lipid profiles and lipid metabolism are at the root of at least some disease mechanisms^21^. Numerous studies have confirmed that some biomarkers may be associated with DN, among which serum lipid levels might play a significant role . Despite the mass of evidence accumulated in the last few years and the considerable contribution of serum lipid profiles to DN, there are still some considerable contradictions regarding the relationship between serum lipid levels and DN in observational and epidemiological studies^23–26^. Some studies have shown a positive association between a high level of total cholesterol (TC) and DN in diabetic patients^27^. In contrast, other studies have found a lack of significant changes in serum lipid profiles or even an inverse association between TG levels and DN^18,28,29^. Ascertaining whether a relation exists between serum lipid profiles and DN might lead to new disease-modifying therapies.

To comprehensively investigate the relationship between DN and the serum levels of TG, TC, high-density lipoprotein cholesterol (HDL), and low-density lipoprotein cholesterol (LDL), we conducted a meta-analysis quantitatively assessing the role of serum lipid levels in DN. The results provide new knowledge regarding the treatment of DN and may help in the development of clinical biomarker guidelines for DN.

## Methods

### Literature search strategy

This meta-analysis was conducted using the PRISMA (Preferred Reporting Items for Systematic Reviews and Meta-Analyses) guidelines^30^. This systematic review was prospectively registered in PROSPERO (CRD42020191400); the registration is available at http://www.crd.york.ac.uk/PROSPERO/display_record.php?ID=CRD42020191400.

Relevant articles were identified through an electronic search of PubMed, Embase, Cochrane Library and Web of Science using the following search terms: (“Diabetic Neuropathy” OR “Neuropathies, Diabetic” OR “Neuropathy, Diabetic” OR “Diabetic Autonomic Neuropathy” OR “Autonomic Neuropathies, Diabetic” OR “Autonomic Neuropathies, Diabetic” OR “Diabetic Autonomic Neuropathies” OR “Neuropathies, Diabetic Autonomic” OR “Neuropathy, Diabetic Autonomic” OR “Diabetic Neuralgia” OR “Diabetic Neuralgias” OR “Neuralgias, Diabetic” OR “Diabetic Neuropathy, Painful” OR “Diabetic Neuropathies, Painful” OR “Neuropathies, Painful Diabetic” OR “Neuropathy, Painful Diabetic” OR “Painful Diabetic Neuropathies” OR “Painful Diabetic Neuropathy” OR “Neuralgia, Diabetic” OR “Symmetric Diabetic Proximal Motor Neuropathy” OR “Asymmetric Diabetic Proximal Motor Neuropathy” OR “Diabetic Asymmetric Polyneuropathy” OR “Asymmetric Polyneuropathies, Diabetic” OR “Asymmetric Polyneuropathy, Diabetic” OR “Diabetic Asymmetric Polyneuropathies” OR “Polyneuropathies, Diabetic Asymmetric” OR “Polyneuropathy, Diabetic Asymmetric” OR “Diabetic Mononeuropathy” OR “Diabetic Mononeuropathies” OR “Mononeuropathies, Diabetic” OR “Mononeuropathy, Diabetic” OR “Diabetic Mononeuropathy Simplex” OR “Diabetic Mononeuropathy Simplices” OR “Mononeuropathy Simplex, Diabetic” OR “Mononeuropathy Simplices, Diabetic” OR “Simplex, Diabetic Mononeuropathy” OR “Simplices, Diabetic Mononeuropathy” OR “Diabetic Amyotrophy” OR “Amyotrophies, Diabetic” OR “Amyotrophy, Diabetic” OR “Diabetic Amyotrophies” OR “Diabetic Polyneuropathy” OR “Diabetic Polyneuropathies” OR “Polyneuropathies, Diabetic” OR “Polyneuropathy, Diabetic”) and (“serum lipid profiles” OR “lipid profiles” OR “lipid levels” OR “triglycerides” OR “total cholesterol” OR “high-density lipoprotein cholesterol” OR “low-density lipoprotein cholesterol”). Relevant articles published up to May 2020 were included in this study. We also manually screened the reference lists of retrieved articles to identify any potentially relevant studies. The summarized search strategy and the full electronic search strategies for multiple international databases are presented in Supplementary Files 2 and 3.

### Selection criteria

Studies were included in this meta-analysis only if any all of the following criteria were met: (1) The study was published as an original article; (2) There were at least 2 groups (a DN group and a healthy control group); (3) The study evaluated the serum levels of TG, TC, HDL, or/and LDL of these 2 groups. Studies were excluded from this meta-analysis if any of the following criteria were met: (1) The study was a review, commentary, case report, case series or letter to the editor; (2) The study was performed in animals or in vitro; (3) The article was not in English (this restriction was imposed because English is the international language of science); (4) There was a significant difference in baseline age, gender, or body mass index (BMI) between the 2 groups. Studies were selected by two reviewers (ZC and YY) for inclusion in our analysis using the aforementioned criteria, and disagreements were resolved by consensus or with the help of a third reviewer (JZ).

### Data extraction and quality assessment

Clinical information was robustly extracted from all eligible studies: number, first author, year, country, N (case/control), age, M%, outcome reported, diabetes and Newcastle–Ottawa scale (NOS) score. Two investigators (ZC and YY) independently extracted study characteristics from the selected studies based on the predetermined inclusion and exclusion criteria. Any disagreements were settled with the help of a third reviewer (JZ) when necessary. For each study, the risk of bias was assessed using the NOS quality assessment instrument, which is used for assessing the quality of nonrandomized studies in a meta-analysis^31^. The measures on this scale comprise three items: the selection of participants, the comparability of cases and controls, and the ascertainment of outcomes. The scale has a minimum score of 0 and a maximum score of 9. Studies scoring at least 7 (corresponding to 78% of the maximum score) were regarded as having a low risk of bias (‘good’ quality); those that scored 4–6 were deemed to have a modest risk of bias (‘fair’ quality); and those that scored < 3 were considered to have a substantial risk of bias (‘poor’ quality)^32^.

### Statistical analysis

Statistical analysis was performed using the STATA software package (version 12.0, STATA Corp, College Station, TX). The results are expressed as mean differences (MDs) and odds ratios (ORs) with 95% confidence intervals (CIs). Lipid levels were extracted as continuous variables for statistical analysis and reported as the mean and standard deviation (SD). We also used the following approximations: if a study provided lipid levels with the mean and standard error, we converted the standard error into an SD by the following equation: standard error × square root of the sample size. If a study provided medians and interquartile ranges, we converted them to means and SDs as described by Hozo et al.^33^. For discrete data, if the OR and 95% CI were not available, a 2 × 2 table was used to obtain the value of OR and 95% CI. A random-effects model was used to calculate the pooled results if the inconsistency index (*I2*) statistic was > 50%, and a fixed-effects model was applied if *I2* ≤ 50%. Data with p ≥ 0.10 and *I2* ≤ 50% were defined as having low heterogeneity. We assessed potential publication bias using funnel plots. Sensitivity analysis was performed by the leave-one-out method to assess whether the results were sufficiently robust and verify that they were not excessively influenced by any single study^34^.

## Results

### Study characteristics

**Figure 1:**
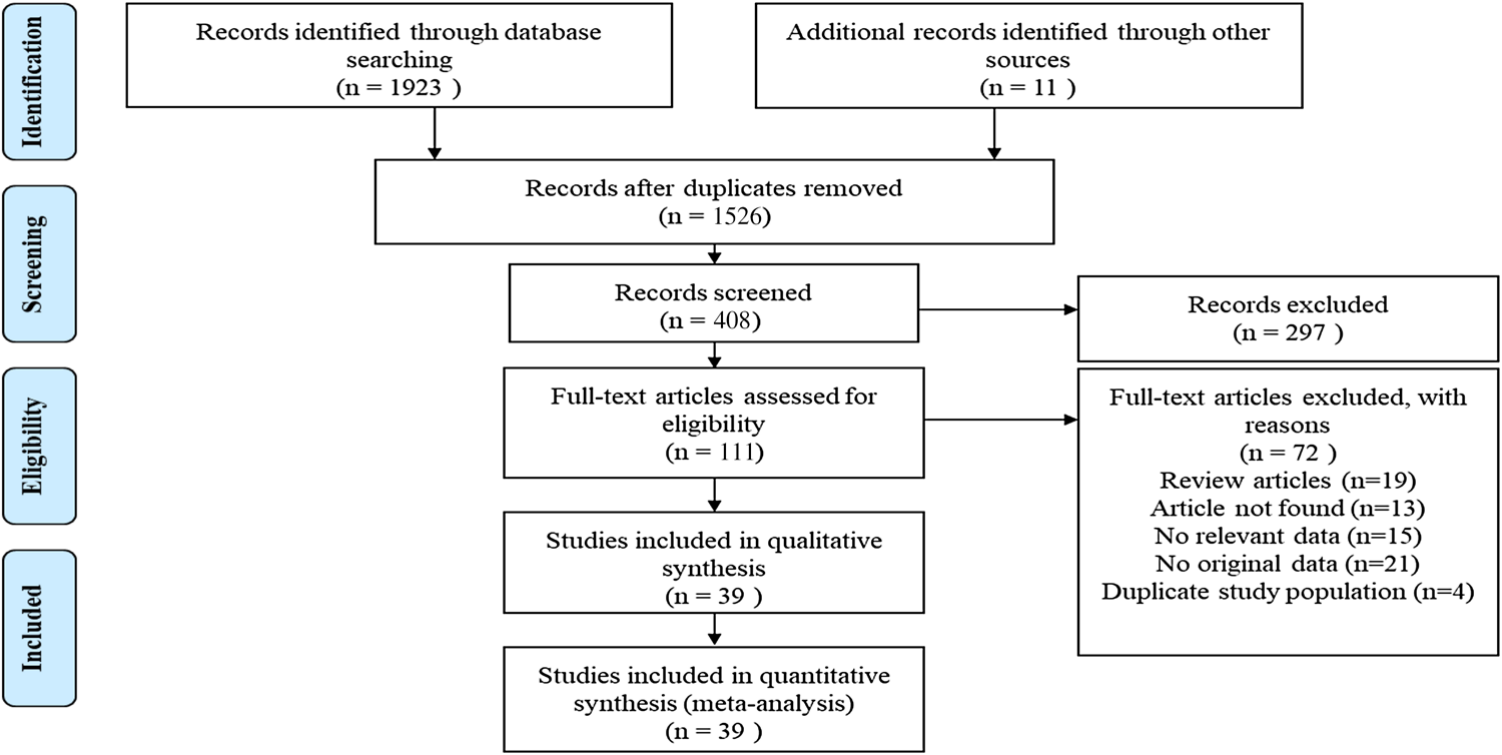
Flow of study selection.

We identified 1923 studies through electronic searches. We also identified and read 11 potentially relevant articles that we found by browsing the reference lists of related articles and reviews. Of the candidate studies, we excluded 1526 after reading the abstracts and titles because they were duplicate studies, review articles, animal studies, commentaries, proceedings, case observations, or irrelevant to the present analysis. By further analysing the full text of the 111 remaining papers, the remaining 39 eligible studies were included in our meta-analysis^18,22–29,35–65^. A flow chart showing our selection process is presented in Fig. 1. Of these articles, 35 studies with 32,198 patients presented data for TG, 29 studies with 22,141 patients reported data for TC, 34 studies with 28,681 patients reported data for HDL and 30 studies with 22,615 patients presented data for LDL. There were 16 studies of patients with T2DM, 10 studies of patients with T1DM, 4 studies of patients with either T1DM or T2DM and 9 studies that did not specify the type of diabetes. Detailed characteristics of these eligible studies are described in Table 1.

**Table 1:** Studies included in the meta-analysis.

Number	References	Country	N (case/control)	Age	M%	Study design	Outcome reported	Diabetes
1	Jende^35^	Germany	100(64/36)	64.6 ± 0.9	68%	cross-sectional cohort	TC, LDL-C, HDL-C, TG	T2DM
2	Hosny^36^	Egypt	60(30/30)	51.238 ± 7.784	NA	a case control study	TC, LDL, HDL, TG	T2DM
3	Song^29^	China	455	62.8 ± 8.61	46%	a case control study	TC,TG,HDL,LDL	DM
4	Vural^37^	Turkey	165(90/75)	64.0 ± 10.3/66.6 ± 14.7	58.90%	retrospective study	TC,TG,LDL-C,HDL-C	DM
5	Andersen^38^	Denmark	144(27/117)	62.59 ± 17.31/52.36 ± 17.52	81.50%	cross-sectional study	TC, LDL-C, HDL-C, TG	T2DM
6	Mizokami-Stout^39^	USA	5936(630/5306)	39 ± 18	45%	cross-sectional study	TG, HDL-C, LDL-C,TC	T1DM
7	Litzelman^40^	African-American 76%	352	60.4 ± 9.6	19%	randomized controlled trial	HDL/cholesterol, HDL,TG,TC	T2DM
8	Aryan^41^	Iran	939(444/495)	43.28 ± 14.42	48%	A nested case–control stud	TG, HDL-C, LDL-C,TC	T2DM
9	Akinci^42^	Turkey	74(31/43)	28.26 ± 10.33/18.26 ± 7.42	12.9%/44.2%	cross-sectional study	TC, TG,LDL-C, HDL-C,	DM
10	Akbar^25^	India	202(62/140)	55.7 ± 10.0/51.3 ± 10.8	33%/61%	cross-sectional cohort	TC,HDL,TG,TyG index	T2DM
11	Aktaş^26^	Turkey	50(27/23)	56.85 ± 9.87/55.39 ± 8.84	7%/10%	cross-sectional cohort	LDL,HDL,TC	DM
12	Hwang^23^	Korea	530(239/291)	20–80	58.5%/51.2%/64.4%/44.3%	retrospective study	TC, TG,LDL-C, HDL-C,	T2DM
13	Jane^43^	China	628	< 65 22.9%/28.1%; ≥ 65 77.1%/71.9%	37%/46.1%	cross-sectional study	TC, TG	T2DM
14	Najafi^44^	Iran	192	58.8 ± 8.2/57.9 ± 8.8	48.9%44.1%	cross-sectional study	TG,TC,LDL-C,HDL-C	T2DM
15	Zoppini^45^	Italy	557(461/96)	58 ± 9.6/56.7 ± 6.6/57.8 ± 9.8	68.75%/66.16%	A cohort	LDL-C, HDL-C, TGs	T2DM
16	Ishibashi^46^	Iapan	107(78/28)	55.7 ± 1.5/54.8 ± 2.2/58.7 ± 2.2/48.1 ± 2.2	54.55%/65.22%	cross-sectional cohort	LDL-C,HDL-C,TG	T2DM
17	Cho^47^	Korea	48(15/33)	2006(55.93 ± 9.23/54.26 ± 10.55);2012(66.60 ± 5.47/63.15 ± 8.81)	NA	retrospective study	TC, TG,LDL-C, HDL-C,	T2DM
18	Rosales-Hernandez^48^	Canada	82(60/12)	61.1 ± 10.0/59.8 ± 10.3/53.6 ± 14.4	69%/64%/50%	a case control study	TC, TG,LDL-C, HDL-C,	DM
19	Jende^49^	Germany	120(84/36)	60.83 ± 1.61/62.55 ± 1.29	60%	cross-sectional cohort	TC, TG,LDL-C, HDL-C,	T1DM and T2DM
20	Smith^22^	USA	218	58.5 ± 9.1/60 ± 7.9/57.5 ± 9.8	50%/53%/55%	retrospective study	TC,LDL,HDL	T2DM
21	Katulanda^50^	Sri Lanka	528	55.0 ± 12.4	0.373	a case control study	TC, TG,LDL-C, HDL-C,	T1DM and T2DM
22	Hsu^24^	China	326(85/241)	65.7 ± 9.3/62.8 ± 9.5	38.8%/31.1%	A cohort	TC, TG	T2DM
23	Hsiao^51^	China	271	55.33 ± 11.04/56.49 ± 6.94	57.93%/60%	cross-sectional study	TC	T2DM
24	Ylitalo^52^	USA	100(9/91)	63.2 ± 0.7/55.8 ± 0.4	65.5%/46.2%	A cohort	TG,LDL-C, HDL-C,	DM
25	Spallone^53^	Italy	191(135/56)	59.9 ± 9.7/57.2 ± 10.5/58.1 ± 9.6	44.87%/71.93%/57.14%	a case control study	TC, TG,LDL-C, HDL-C,	T1DM and T2DM
26	Terekeci^65^	USA	42(25/17)	58.80 ± 8.60/55.18 ± 6.41	50%	A cohort	TC,TG	T2DM
27	Faisal^27^	Bahrain	1225(526/689)	54/52	42.99%	cross-sectional cohort	TC,TG,HDL-C	T1DM and T2DM
28	Coppini^55^	UK	300(100/200)	66.2 ± 9.4 /49.2 ± 16.3	Gender (M:F) 1.3 : 1 /2 : 1	a retrospective case–control study	TC, TG,LDL-C, HDL-C,	DM
29	Kempler^28^	31 centres in 16 European countries	3270	32.7 ± 10.2	51.62%	A cohort	TC, TG,LDL-C, HDL-C,	T1DM
30	Christen^64^	USA	407	31.4 ± 1.4	75.30%	a prospective cohort study	LDL,HDL,TG	T1DM
31	Maser^57^	Pittsburgh	363(228/135)	34 ± 6/28 ± 6	52%/50%	A cohort	LDL,HDL,TG	T1DM
32	Maser^58^	Pittsburgh	168(105/63)	30 ± 3/29 ± 3	49%/57%	A cohort	LDL,HDL,TG	T1DM
33	Orchard^59^	Pittsburgh	325(57/268)	< 17	47%/56%	A cohort	TC,TG,LDL-C, HDL-C,	T1DM
34	Tesfaye^18^	31 centres in 16 European countries	3250	NA	51.32%	A cohort	LDL,HDL,VLDL	T1DM
35	Simmons^60^	UK	33(20/13)	49 ± 5/50 ± 11/50 ± 6	63.64%/66.67%/46.15%	cross-sectional cohort	TC, TG	T1DM
36	Tesfaye^9^	31 centers in the European Diabetes	1172(276/896)	29.8 ± 8.1/33.6 ± 10.0	51.1%/48.6%	prospective study	TC, TG,LDL-C, HDL-C,	T1DM
37	Witte^56^	UK	956(163/793)	34.5 ± 10.3/30.7 ± 8.4	35.58%/6.68%	A cohort	TC, TG,LDL, HDL	T1DM
38	Callaghan^62^	USA	2382	73.5 ± 2.9	48.30%	A cohort	TG,HDL	DM
39	Callaghan^63^	China	4002	51.6 ± 11.8	51%	A cross-sectional	TG,HDL	DM

### Quality assessment

All thirty-nine studies had NOS quality scores greater than or equal to 5, indicating that all these studies had ‘good’ or ‘fair’ methodological quality. Details on the risk of bias among those 39 studies are summarized in Table 2.

**Table 2:** Risk of bias analysis in each study.

Number	References	Selection	Comparability	Outcome ascertainment	Bias risk (Total scores)	Final quality conclusion
1	Jende^35^	4	1	1	6	Fair
2	Hosny^36^	4	2	1	7	Good
3	Song^29^	3	2	1	6	Fair
4	Vural^37^	2	2	1	5	Fair
5	Andersen^38^	3	2	3	8	Good
6	Mizokami-Stout^39^	3	2	3	8	Good
7	Litzelman^40^	4	2	2	8	Good
8	Aryan^41^	3	2	2	7	Good
9	Akinci^42^	2	2	1	5	Fair
10	Akbar^25^	3	1	2	6	Fair
11	Aktaş^26^	2	1	2	5	Fair
12	Hwang^23^	4	2	1	7	Good
13	Jane^43^	4	1	3	8	Good
14	Najafi^44^	3	1	2	6	Fair
15	Zoppini^45^	4	2	3	9	Good
16	Ishibashi^46^	2	1	2	5	Fair
17	Cho^47^	2	1	2	5	Fair
18	Rosales-Hernandez^48^	3	1	1	5	Fair
19	Jende^49^	3	2	1	6	Fair
20	Smith^22^	3	2	2	7	Good
21	Katulanda^50^	4	2	2	8	Good
22	Hsu^24^	4	2	1	7	Good
23	Hsiao^51^	4	3	1	8	Good
24	Ylitalo^52^	3	2	1	6	Fair
25	Spallone^53^	3	1	2	6	Fair
26	Terekeci^65^	2	2	1	5	Fair
27	Faisal^27^	4	2	3	9	Good
28	Coppini^55^	3	2	3	8	Good
29	Kempler^28^	4	2	3	9	Good
30	Christen^64^	4	1	1	6	Fair
31	Maser^57^	3	2	2	7	Good
32	Maser^58^	3	1	1	5	Fair
33	Orchard^59^	3	1	2	6	Fair
34	Tesfaye^18^	4	2	3	9	Good
35	Simmons^60^	3	1	1	5	Fair
36	Tesfaye^9^	4	2	3	9	Good
37	Witte^56^	3	2	3	8	Good
38	Callaghan^62^	4	2	3	9	Good
39	Callaghan^63^	4	2	3	9	Good

### Serum TG levels between DN and non-DN patients/healthy controls

**Figure 2:**
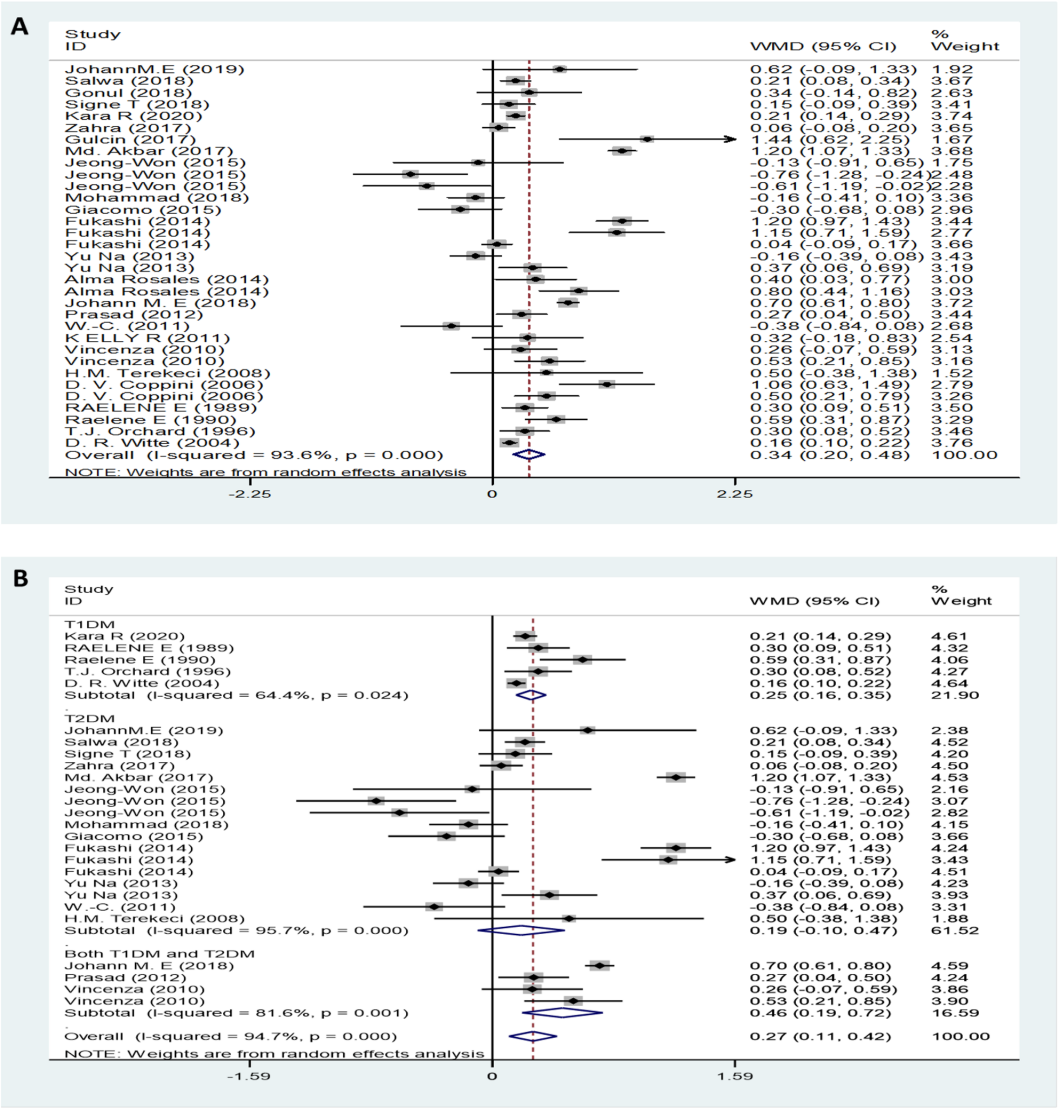

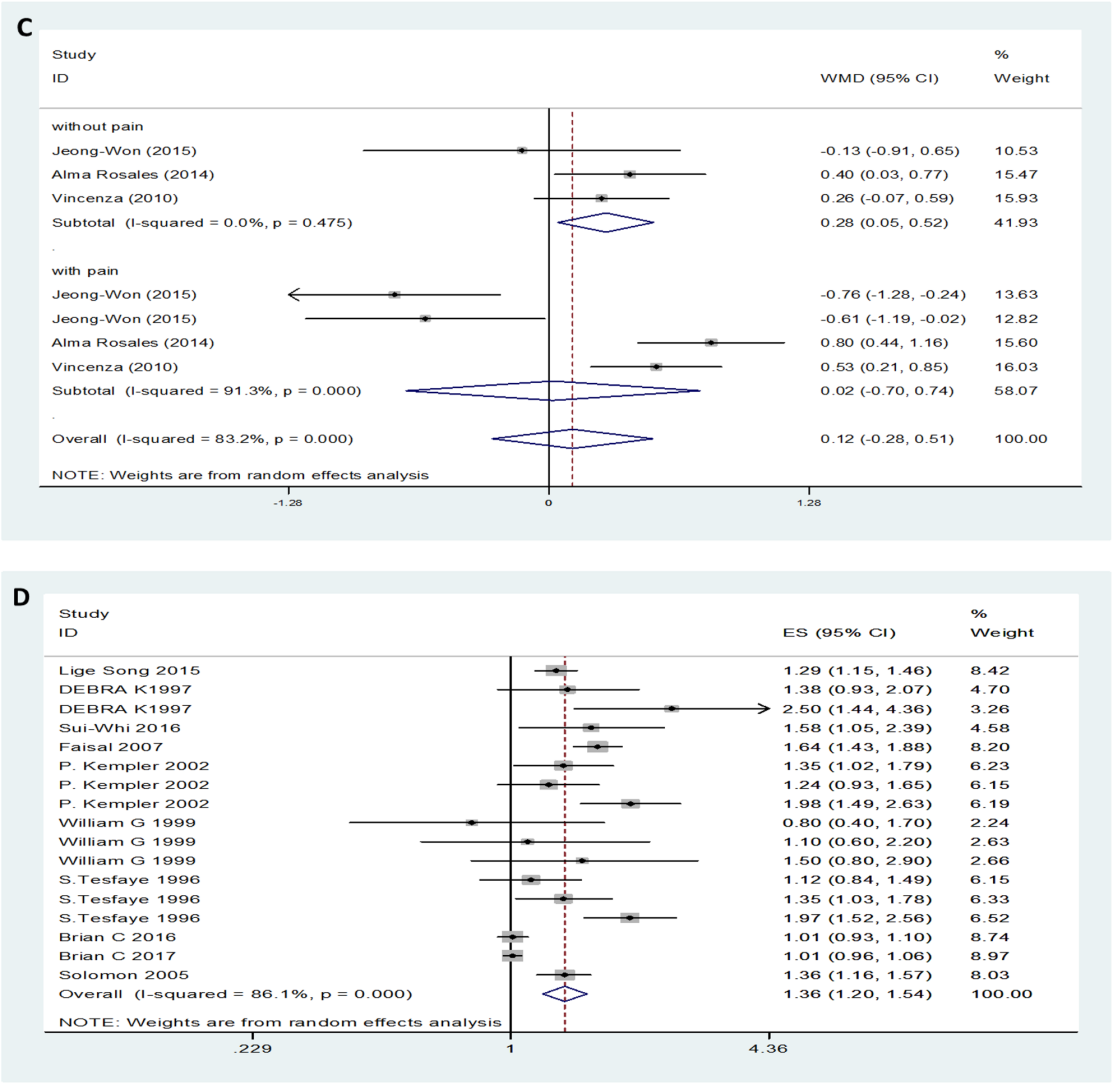
TG levels (**A**) in people with DN versus those without DN. TG levels in the subgroup analysis stratified by the type of diabetes (**B**) and symptom severity (**C**). OR (**D**) for DN in patients according to serum TG levels.

The pooled TG results with of 35 studies on TG showed a significantly increased serum TG levels in DN patients compared to non-DN patients with a random-effects model (MD (95% CI): 0.34 (0.20–0.48), *I*^*2*^ = 93.6%, p < 0.001) (Fig. 2A). Moreover, the serum TG levels of neuropathy patients with T1DM were higher than those of control patients (Fig. 2B). Patients with moderate or severe pain had no significant difference in TG levels of TG than compared with patients with mild or painless controls patients (MD (95% CI): 0.12 (-0.28– 0.51), *I*^*2*^ = 83.2%, p < 0.001) (Fig. 2C). Furthermore, compared with the lower serum TG level category, the highest serum TG level showed an increased the risk of DN (OR (95% CI): 1.36 (1.20–1.54), *I*^*2*^ = 86.1%, p < 0.001) (Fig. 2D).

### Serum TC between DN and non-DN patients

**Figure 3:**
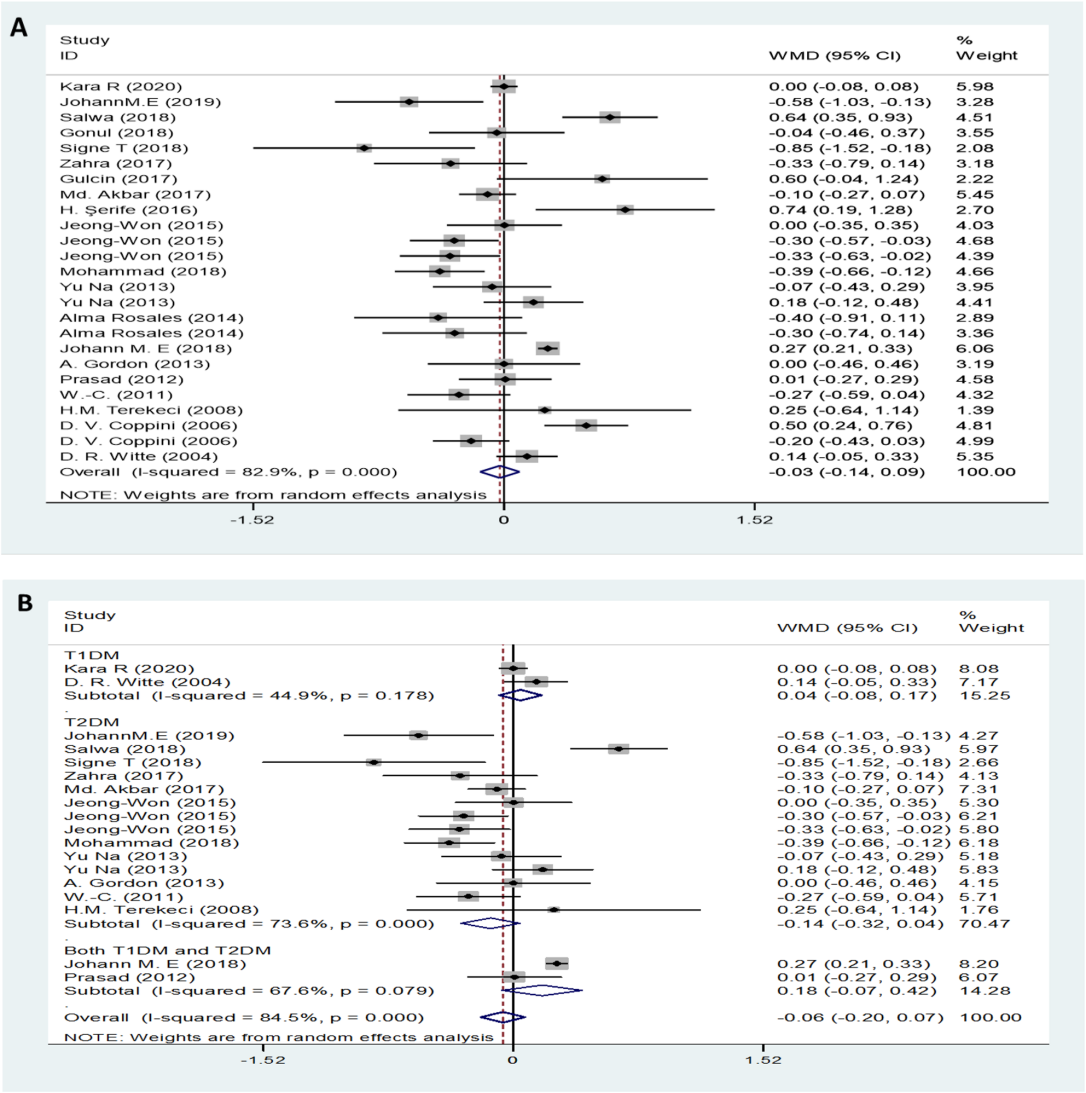

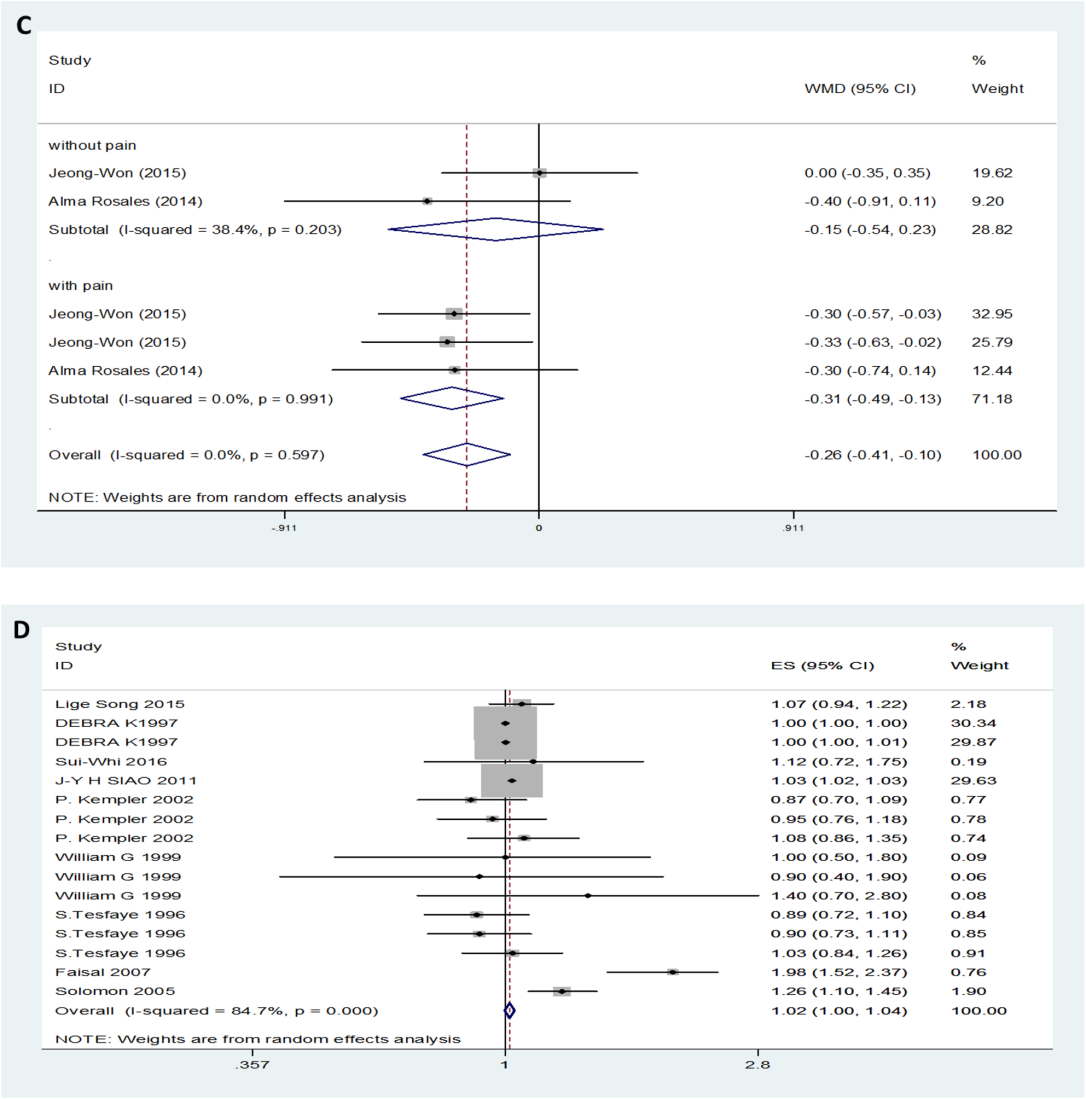
TC levels (**A**) in people with DN versus those without DN. TC levels in the subgroup analysis stratified by the type of diabetes (**B**) and symptom severity (**C**). OR (**D**) for DN in patients according to serum TC levels.

Twenty-nine studies, provided data on TC. Forest plot indicated that the expression level of TC in DN patients was not significantly different from that in control patients with a random-effects model (MD (95% CI): -0.03 (-0.14–0.09), *I*^*2*^ = 82.9%; p < 0.001) (Fig. 3A). The expression levels of TC in both T1DM neuropathy and T2DM neuropathy were not significantly different from those in the control group (Fig. 3B). Additionally, for symptomatic DN, moderate or severe DN patients had lower levels of serum TC compared to mild or painless DN patients (MD (95% CI): -0.31 (-0.49– -0.31), *I*^*2*^ = 0%, p = 0.991) (Fig. 3C). Patients with high levels of TC level had no influence on the risk of DN (OR (95% CI): 1.02 (1.00–1.04), and there was obvious evidence of significant heterogeneity among studies (*I*^*2*^ = 84.7%, p < 0.001) (Fig. 3D).

### Serum HDL levels between of DN and non-DN patients/healthy controls

**Figure 4:**
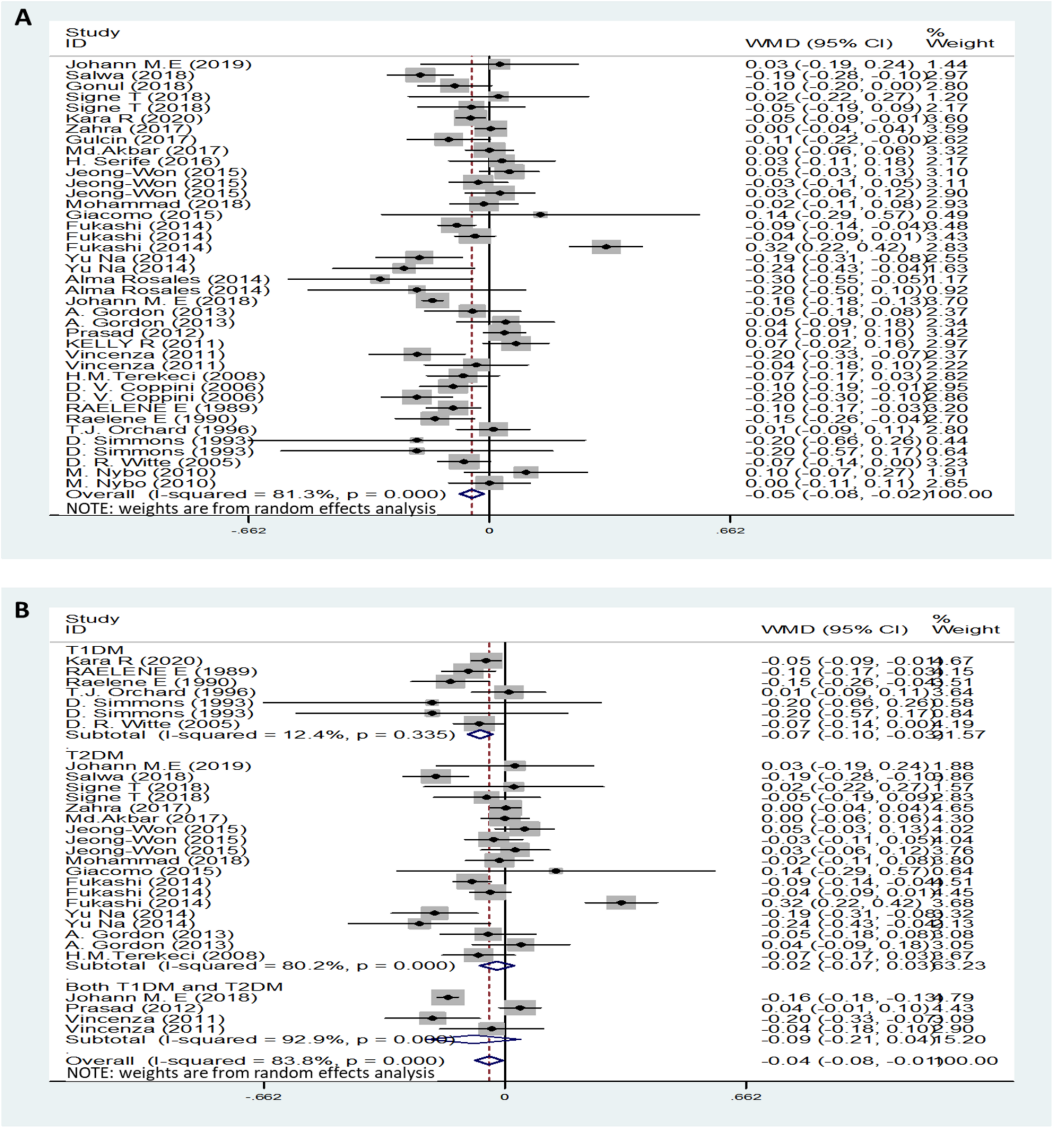

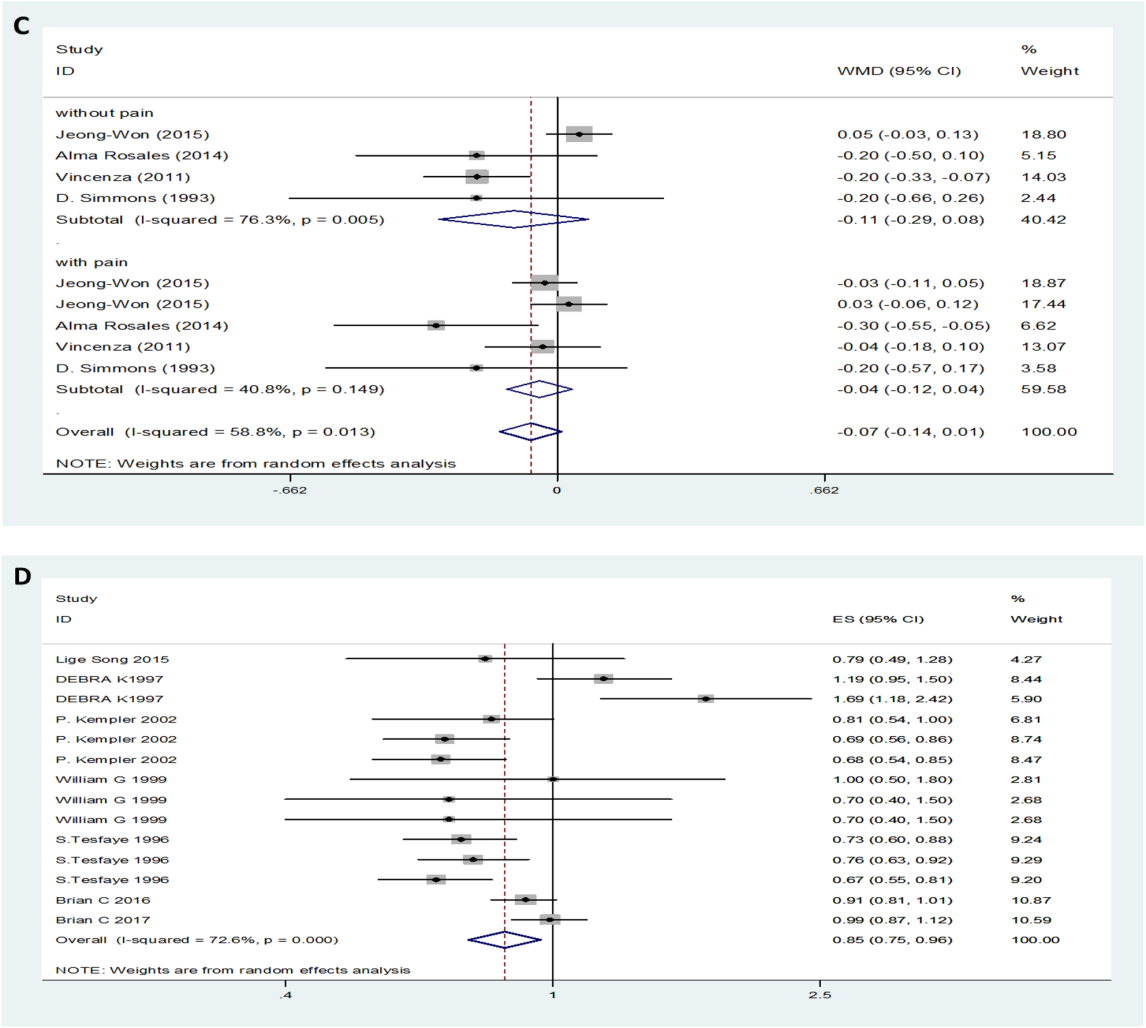
HDL levels (**A**) in people with DN versus those without DN. HDL levels in the subgroup analysis stratified by the type of diabetes (**B**) and symptom severity (**C**). OR (**D**) for DN in patients according to serum HDL levels.

Data on HDL levels for DN were obtained from 34 studies. The results showed that serum HDL levels in patients with DN were lower than those in the control group with a random-effects model (MD (95% CI): -0.05 (-0.08‐ -0.02, *I*^*2*^ = 81.3%; p < 0.001) (Fig. 4A). Interestingly, subgroup analysis found that only patients with T1DM neuropathy had lower HDL levels than patients in the control group, while serum HDL levels in patients with T2DM were not different from those in the control group (Fig. 4B). The change in the MD for serum HDL levels was not significantly different between DPN and painless neuropathy (MD (95% CI): -0.07 (-0.04–0.01), *I*^*2*^ = 58.8%, p = 0.013 (Fig. 4C). In addition, high levels of HDL were observed to decrease the risk of DN (OR (95% CI):0.85 (0.75–0.96), *I*^*2*^ = 72.6%, p < 0.001 (Fig. 4D).

### Serum LDL levels between DN and non-DN patients/healthy controls

**Figure 5:**
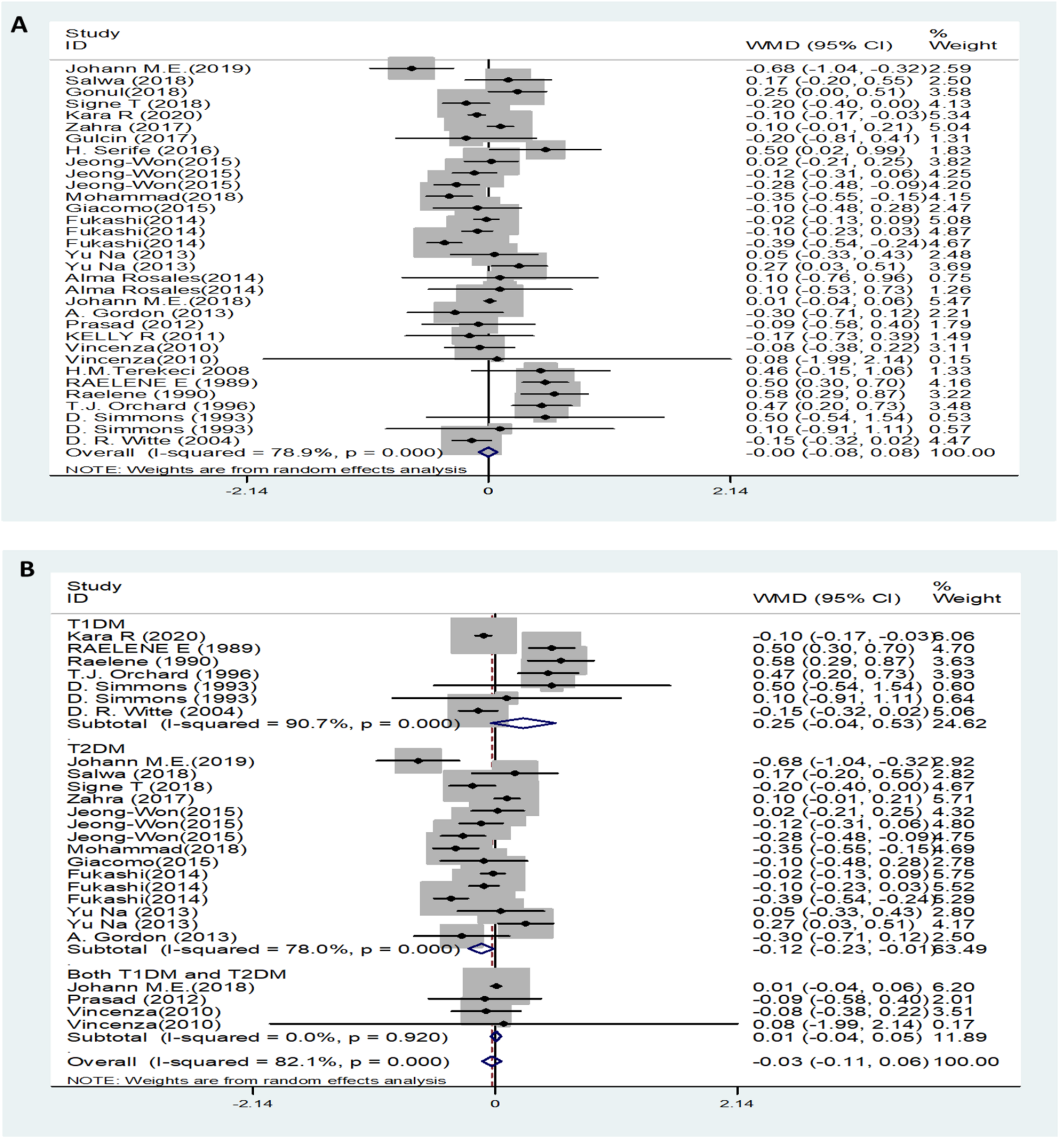

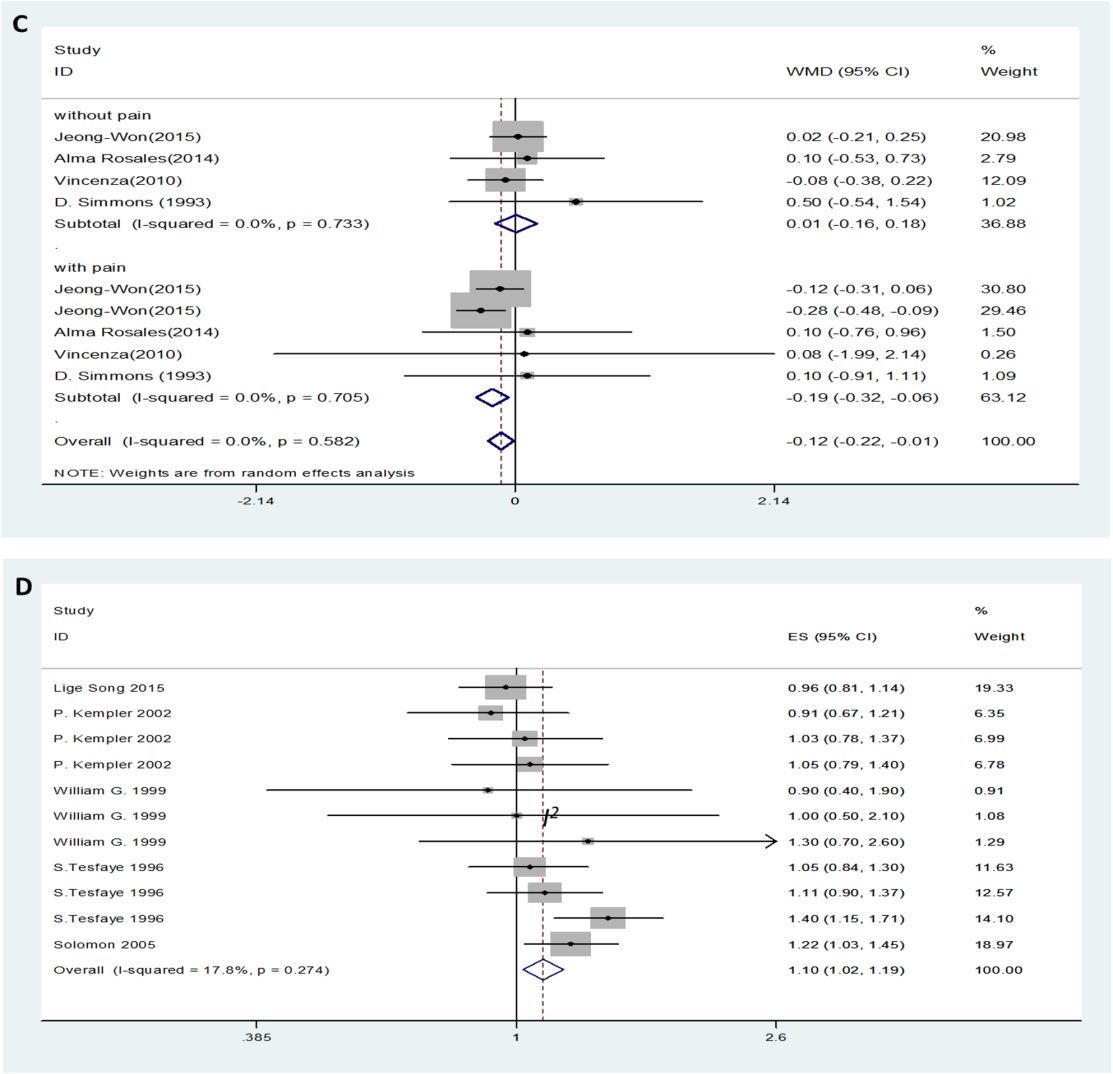
LDL levels (**A**) in people with DN versus those without DN. LDL levels in the subgroup analysis stratified by the type of diabetes (**B**) and symptom severity (**C**). OR (**D**) for DN in patients according to serum LDL levels.

Twenty-nine trials showed no effect of LDL levels on DN. DN patients showed no difference in LDL levels compared to the control group with the random-effects model (MD (95% CI): -0.00 (-0.08–0.08, *I*^*2*^ = 78.9%; p < 0.001) (Fig. 5A). There was no significant difference in LDL levels in either type 1 or type 2 diabetic neuropathy patients compared with the control group (Fig. 5B). Moderate or severe pain DN patients had lower levels of LDL compared to than mild or painless DN patients (MD (95% CI): − 0.19 (− 0.32‐ − 0.06), *I*^*2*^ = 0%, p = 0.705) (Fig. 5C). Serum LDL levels increased the risk of DN (OR (95% CI): 1.10 (1.02‐1.19, *I*^*2*^ = 17.8%, p = 0.274) (Fig. 5D).

### Sensitivity analyses and publication bias

**Figure 6:**
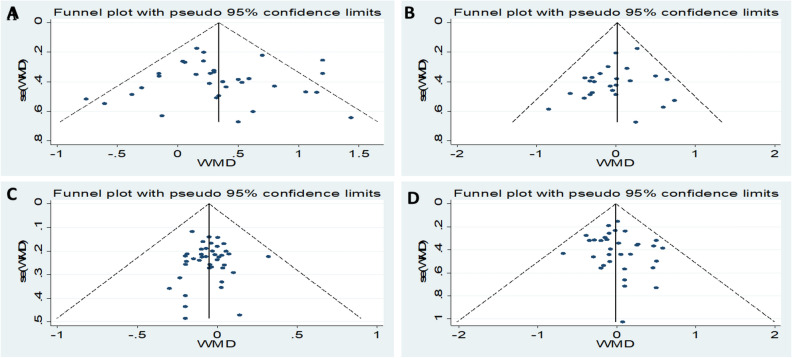
Publication bias funnel plots of the MD for (**A**) TG, (**B**) TC, (**C**) HDL, and (**D**) LDL and DN.

**Figure 7:**
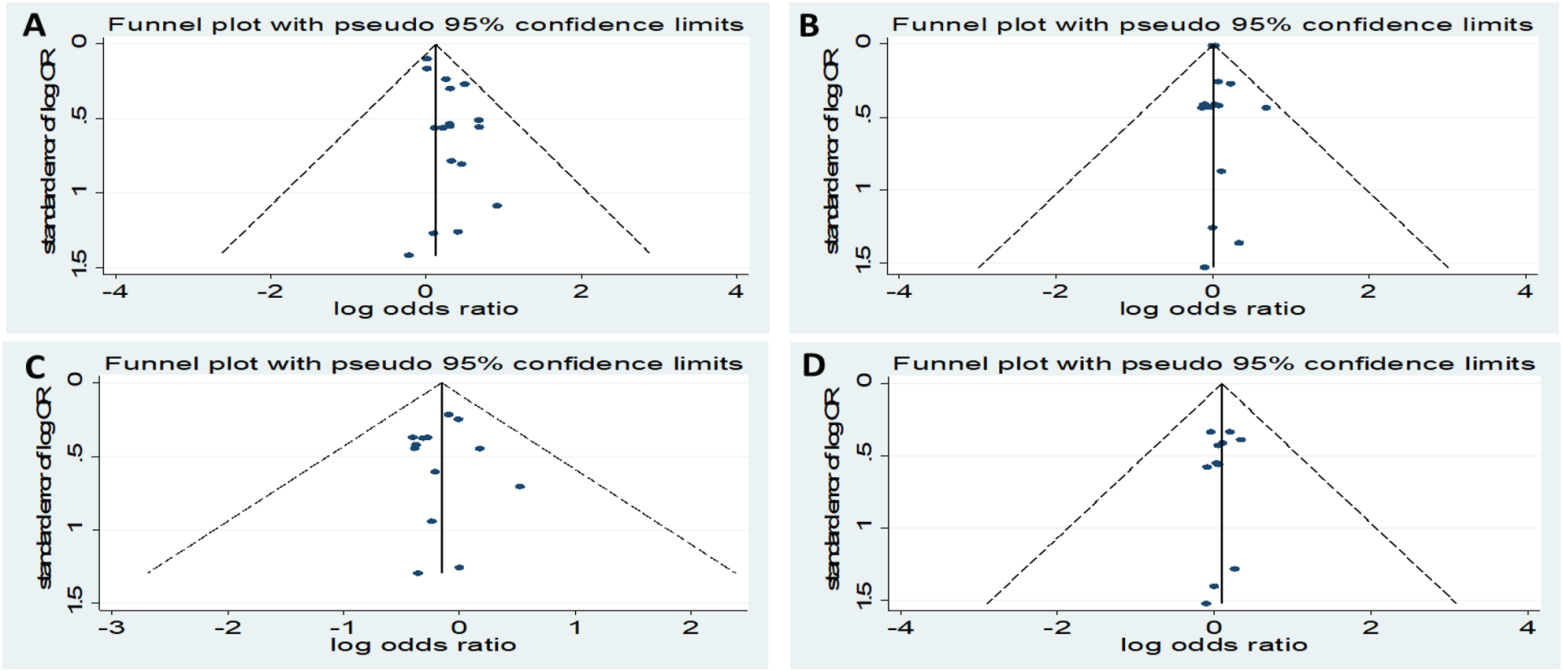
Publication bias funnel plots of the OR for (**A**) TG, (**B**) TC, (**C**) HDL, and (**D**) LDL and DN.

**Figure 8:**
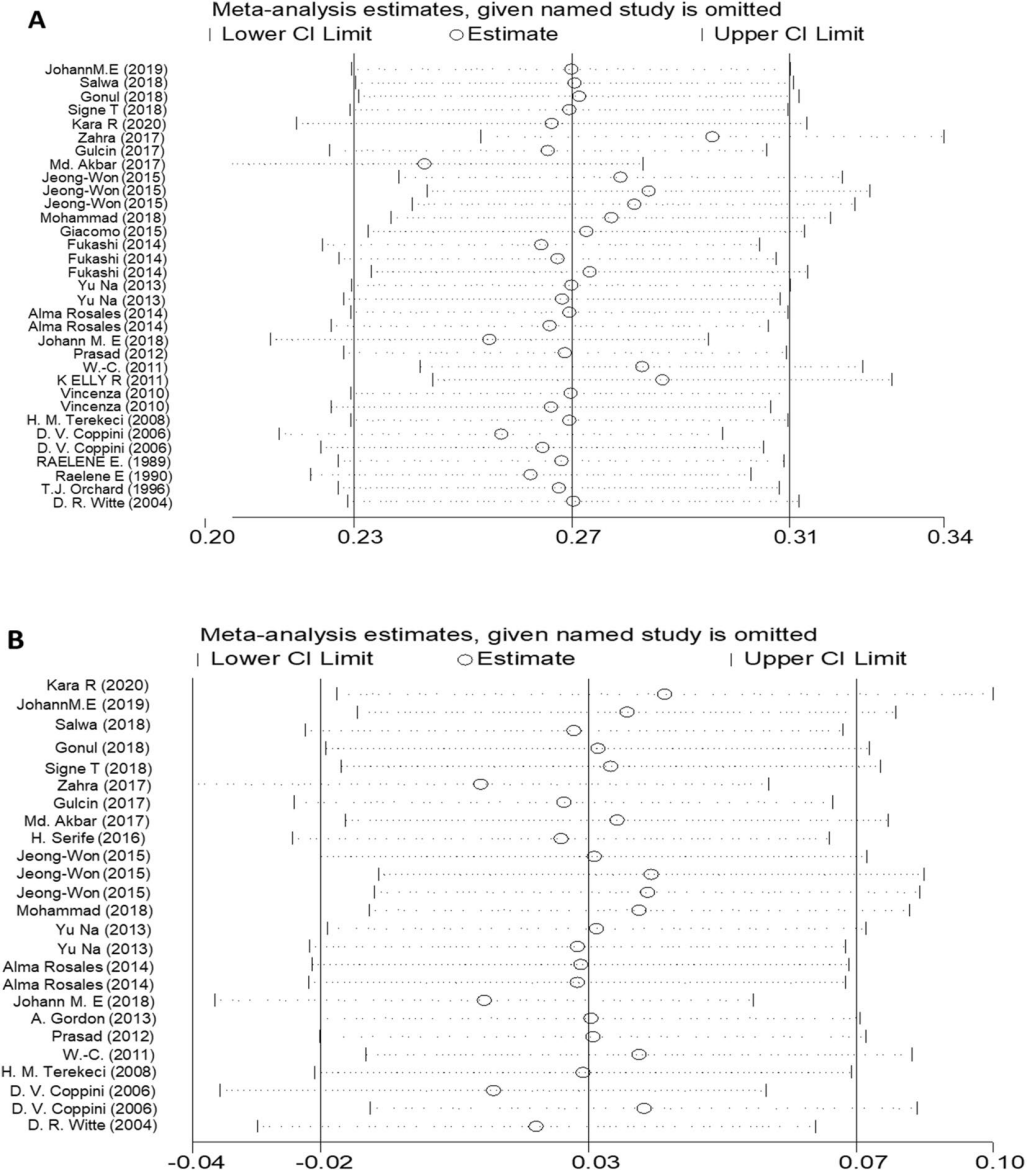

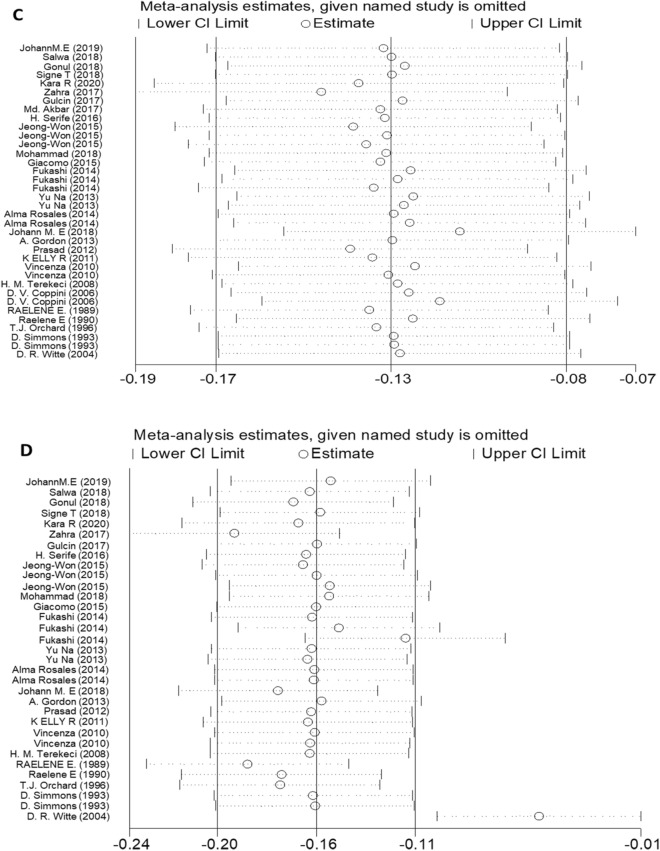
Sensitivity analysis of the pooled MD for (**A**) TG, (**B**) TC, (**C**) HDL, and (**D**) LDL.

**Figure 9:**
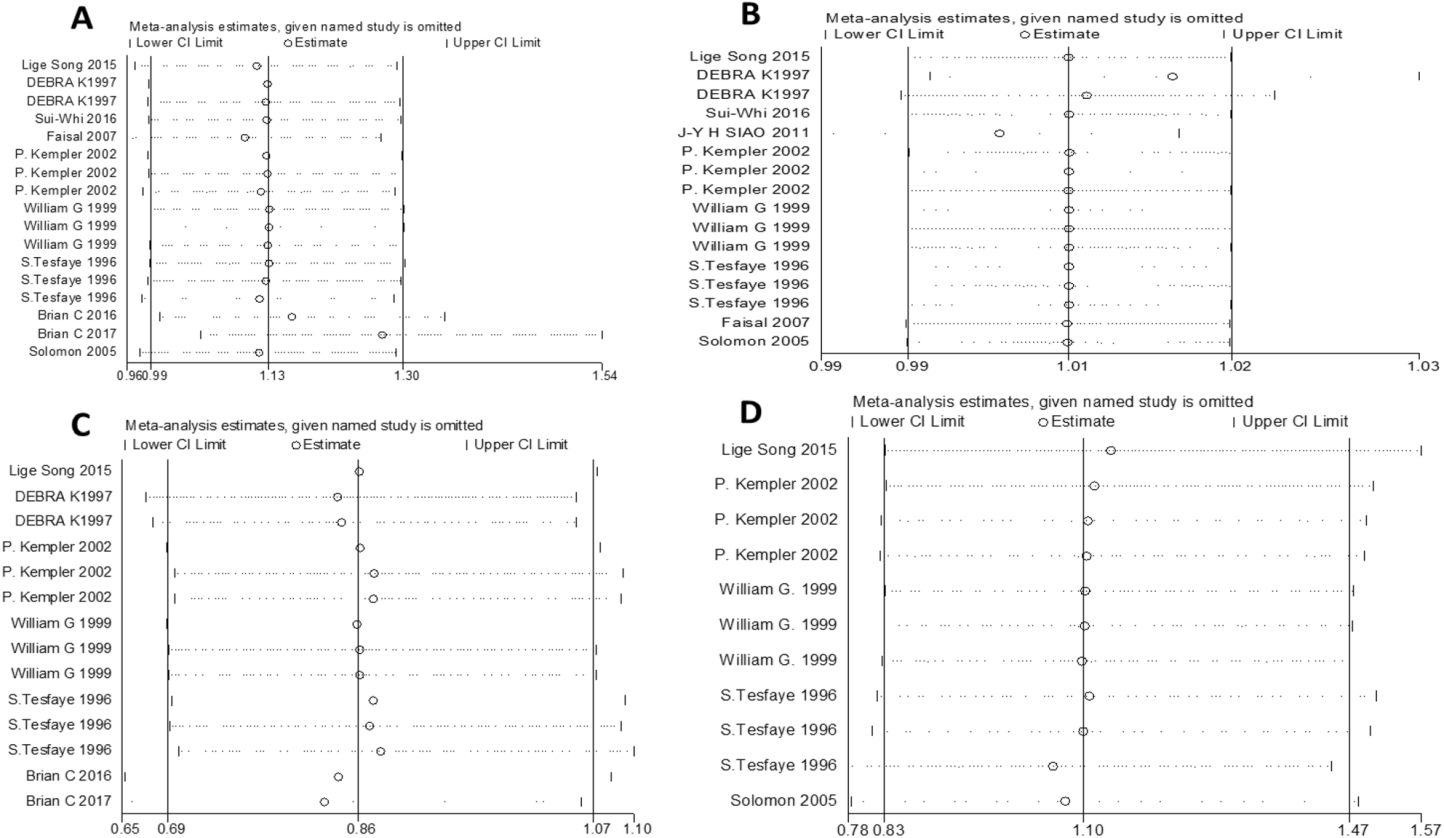
Sensitivity analysis of the pooled OR for (**A**) TG, (**B**) TC, (**C**) HDL, and (**D**) LDL.

Visual inspection of the funnel plot did not reveal remarkable asymmetry (Figs. 6, 7). Sensitivity analysis was performed to analyse the pooled results of the remaining studies by sequential removal of individual studies. There was no significant change in the overall outcomes after removing any single study, suggesting the stability and reliability of our results and that the data were not influenced by any given study (Figs. 8, 9). Thus, the above results suggest that publication bias was not apparent in this meta-analysis.

## Discussion

DN is a common cause of morbidity and death among patients with diabetes^66^; this form of neuropathy is characterized by pain, paraesthesia, sensory loss, an increased frequency of falls, and reduced quality of life (QOL)^9,67^. It is obvious that DN poses a heavy health challenge to individuals. The duration and level of hyperglycaemia are important determinants of diabetic complications, including DN^68^. The risk of DN can be reduced with intensive blood glucose control in T1DM patients. Intensive blood glucose control has little influence in patients with T2DM^15–17^. One study showed that DN remained substantial, despite intensive control of the glucose level^68^. Moreover, except for optimal glycaemic control, there have been no definite positive prevention studies of other risk factor modifications for DN. Thus, there may be risk factors aside from hyperglycaemia involved in the development of DN. Identifying them, particularly if they are modifiable, might lead to new risk-reduction strategies. Accumulated evidence has shown a correlation between DN and serum lipid profiles but has shown inconsistent results^18,28,29,40^. Here, we performed a systematic review and meta-analysis and found that serum lipid profile changes are correlated with DN. To our knowledge, this is the first meta-analysis to provide evidence of a close relationship between TG, TC, HDL, LDL levels and DN risk.

### Exploration of heterogeneity

Because of the apparent heterogeneity of our study, subgroup analysis was performed. We investigated whether the different diabetic types (T1DM or T2DM) and different symptoms (mild and painless or moderate and severe pain) affected the results. These factors may partly explain the origin of heterogeneity.

On the basis of the results, we performed a subgroup analysis with the different diabetic types. In the pooled analysis of the studies, regardless of T1DM or T2DM, the trend of TG change was consistent, and the TG levels of patients with DN were increased compared with those of the control group (Fig. 2B). However, serum HDL levels decreased only in T1DM neuropathy patients, and there was no difference between the control group patients and T2DM neuropathy patients (Fig. 4B). Furthermore, this approach did not obviously change the heterogeneity between the individual efficacy estimates. In addition, another subgroup was performed regarding the different symptoms. Heterogeneity was significantly reduced when analyses were stratified by different symptoms (Fig. 2C, 3C and 5C). We also separately analysed location and study design, as these were assumed to be potential sources of bias (data not shown). These methods slightly change the heterogeneity between the individual efficacy estimates, although they do not eliminate it.

Due to the limited number of included studies, subgroup analysis could not be conducted to explore the heterogeneity of some outcomes. Other uncharacteristic or unexplained underlying factors may contribute to the heterogeneity.

### Relationship between lipid profile and DN

Changes in lipid levels are obvious biological characteristics of DN. After pooled data from 39 studies, our results showed that DN patients had higher levels of serum TG than control patients (Fig. 2A), while HDL levels were lower in DN patients than in control patients (Fig. 4A). The above results indicated that serum detection by TG and HDL may be serological markers for diabetic patients with or without DN. Patients with moderate or severe pain had lower levels of TC and LDL compared to mild or painless DN patients (Figs. 3C and Fig. 5C). It is worth mentioning that TC and LDL level changes in serum may be markers of symptomatic diabetes neuropathy. In addition, the results of our meta-analysis showed that higher levels of TG and LDL significantly increased the risk of DN (Fig. 2D and Fig. 5D). These findings suggest that changes in lipid profiles, especially TG and LDL serum levels, may be risk factors for DN. High-quality randomized controlled trials (RCTs) in the future are needed to better understand the causality between the serum lipid profile and DN.

### Underlying mechanisms of lipid effects on DN

There is a high incidence of dyslipidaemia in both T1DM and T2DM patients, and dyslipidaemia is linked to DN (Figs. 2, 3, 4, 5). The mechanisms by which plasma lipids influence DN have not been fully elucidated, but certain factors may be involved. First, patients with dyslipidaemia are characterized by insulin resistance and a chronic inflammation status^69^ that can also contribute to insulin resistance^70^. Furthermore, insulin resistance has shown a positive association with peripheral neuropathy^71^.

Second, oxidative stress is an important risk factor for DN. Neurons express scavenger receptors for oxidized LDLs, such as oxidized LDL receptor 1^72^. Elevated LDL has increased susceptibility to oxidation and oxidized LDLs (oxLDLs), and these modified LDLs can bind to extracellular receptors, triggering signalling cascades that activate oxidative stress. oxLDL-induced oxidative stress has been shown to mediate nerve injury in a mouse model of dyslipidaemia-induced neuropathy^73^. In addition, oxLDL is involved in neuron injury through nicotinamide adenine dinucleotide phosphate (NADPH) oxidase activation, which leads to increased superoxide production^73^. Additionally, free fatty acids (FFAs) bind to excess intramitochondrial pyruvate, leading to the production of reactive oxygen species. FFAs have been shown to directly cause damage to Schwann cells in vitro, and they can also cause proinflammatory factors to be released from adipocytes and macrophages^71^.

Third, demyelination due to lipid profile disorders is another potential mechanism for lipid-induced nerve injury. Segmental demyelination is an important feature of DN patients, and myelin breakdown with focal demyelination has been shown to occur in high-fat fed mice^74^. Thus, it is plausible to suggest that dyslipidaemia negatively impacts myelination status in nerves and contributes to the development of DN. Moreover, some studies have indicated that TC can be oxidized to oxysterols, which have been shown to lead to neuronal apoptosis^72,75^.

Of these, insulin resistance, inflammation, oxidative stress, and demyelination are possible mechanisms linking lipid profile disorder to DN.

Surprisingly, TC and LDL serum levels were reduced in patients with severe pain compared with asymptomatic conditions. However, at present, there is no reasonable explanation to explain this phenomenon.

### Why intensive blood glucose control has little influence in patients with T2DM

Hyperglycaemia is a key factor underlying DN, but other changes also contribute, including dyslipidaemia and changes in insulin signalling^17^.

Patients with type 2 diabetes have an elevated incidence of dyslipidaemia, which is associated with the occurrence of DN. A study demonstrated that obesity, LDL, HDL, and hypertriglyceridemia were independently associated with neuropathy^17^. A series of bioinformatics analyses identified 532 differentially expressed genes between patient samples with progressive versus nonprogressive diabetic polyneuropathy and found that these were functionally enriched in pathways involving lipid metabolism and inflammatory responses^76^.

T2DM patients are insulin resistant. Disruption of insulin signalling due to insulin resistance makes neurons more vulnerable to metabolic insults and may contribute to the development of DN^77^.

Hyperglycaemia and dyslipidaemia, together with altered insulin signalling, lead to several pathological alterations in neurons, glia and vascular cells, which can lead to nerve dysfunction and ultimately DN.

### Theoretical and practical implications

Our meta-analysis provides good guidance for clinical and scientific research. The treatment of DN has largely been directed at the control of symptoms and glucose control rather than to treat the underlying mechanisms^17^. To date, clinical interventions to treat DN have mainly focused on glycaemic control, while even proper control of blood sugar is a poor efficacy for T2DM neuropathy patients^17^. Along with substantial research into the relationship between serum lipid profiles and DN, the results of our present meta-analytic investigation indicate a clear direction: high levels of TG and LDL increased the risk of DN. In addition, DN patients had higher serum TG and lower HDL levels. Therefore, routine examination of serum TG and HDL levels in diabetic patients to predict the risk of DN is essential. Elevated levels of TG and low levels of HDL may be biomarkers for DN, except for the influence of lifestyle and other factors on blood lipid levels. The clinical use of lipid-lowering drugs may be an effective way to prevent and treat DN. PDN is associated with considerable morbidity, mortality and diminished quality of life^9^. The results of our meta-analysis showed that the presence of decreased TC and LDL in patients with DN may indicate a transition from asymptomatic status to severe pain status in DN patients. To the best of our knowledge, this is the first meta-analysis to explore the relationship between DN and serum lipid profiles; the results may be helpful for future clinical diagnosis and treatment. Blood lipid profiles are an appropriate source of biomarkers for DN screening; additionally, these profiles are widely applicable and easily measured.

The implication of our meta-analysis for scientific research is as follows: first, since the included primary studies had different cut-off values of serum lipids, we strongly suggest that the cut-off value be made uniform in subsequent studies. Second, blood lipid levels can be affected by many conditions, such as the environment, lifestyle and diet, and more detailed characteristics of patients should be recorded. Third, TG, TC, HDL and LDL levels provided a state of lipid levels. However, the other parameters may be considered; for instance, TC/HDL and LDL/HDL ratios are also considered indexes for the prediction of DN. Finally, identifying the mechanism of DN is the basis of treating DN.

### Limitations of our study

Our current meta-analysis provides stable evidence of the relationship between the serum lipid profile and DN. Several limitations should be recognized. First, we found significant heterogeneity in the relationship between serum lipid profile and DN risk, which might result from a very large number of included studies and differences in study quality and basic participant characteristics. Second, the mechanisms underlying the decline in serum TC and LDL levels in moderate or severe pain are unclear. Third, there is a shortage of RCTs investigating the impact of lipid levels on DN risk. Thus, in order to obtain a more precise assessment of the impact of lipids on DN risk, well-designed RCTs are necessary. Fourth, DN represents a heterogeneous syndrome, and various definitions and methods are used to formulate the diagnosis. Screening tests for subjective symptoms or objective instrumental assessments, such as vibration perception threshold measurement, are used in the diagnosis of DN^74^. This increases the heterogeneity between studies. Finally, the included studies were all non-RCTs; thus, causality cannot be shown.

## Conclusion

This meta-analysis supports that higher TG and lower HDL levels may be markers for predicting the development of DN in diabetic patients. Higher TG and LDL levels increase the risk of DN. Reduced TC and LDL serum levels in patients with DN may be a marker of an asymptomatic condition to severe pain condition in patients with DN. The mechanisms underlying the effect of serum TG and LDL levels on the increased risk of DN in recent tobacco quitters need to be further explored, since an improved understanding of this effect could contribute to the development of targeted pharmaceuticals that could be used to aid lipid profile regulation and prediction or treatment of DN.

## Supplementary Information

Supplementary Information 1.

Supplementary Information 2.

Supplementary Information 3.
