# Supplementary material for: A systematic review and meta-analysis of the serum lipid profile in prediction of diabetic neuropathy
Source: Sci Rep. 2021 Jan 12;11:499. doi: 10.1038/s41598-020-79276-0 (PMC7804465; doi:10.1038/s41598-020-79276-0)
Supplement: Supplementary file 1 — Supplementary Information 1. [file 41598_2020_79276_MOESM1_ESM.docx]

The PRISMA for Abstracts Checklist

| **TITLE** | **CHECKLIST ITEM** | REPORTED ON PAGE # |
| --- | --- | --- |
| 1. Title: | Identify the report as a systematic review, meta-analysis, or both. | 1 |
| **BACKGROUND** |  |  |
| 2. Objectives: | The research question including components such as participants, interventions, comparators, and outcomes. | 4-6 |
| **METHODS** |  |  |
| 3. Eligibility criteria: | Study and report characteristics used as criteria for inclusion. | 6-9 |
| 4. Information sources: | Key databases searched and search dates. |  |
| 5. Risk of bias: | Methods of assessing risk of bias. |  |
| **RESULTS** |  |  |
| 6. Included studies: | Number and type of included studies and participants and relevant characteristics of studies. | 9-12 |
| 7. Synthesis of results: | Results for main outcomes (benefits and harms), preferably indicating the number of studies and participants for each. If meta-analysis was done, include summary measures and confidence intervals. |  |
| 8. Description of the effect: | Direction of the effect (i.e. which group is favoured) and size of the effect in terms meaningful to clinicians and patients. |  |
| **DISCUSSION** |  |  |
| 9. Strengths and Limitations of evidence: | Brief summary of strengths and limitations of evidence (e.g. inconsistency, imprecision, indirectness, or risk of bias, other supporting or conflicting evidence) | 12-18 |
| 10. Interpretation: | General interpretation of the results and important implications |  |
| **OTHER** |  |  |
| 11. Funding: | Primary source of funding for the review. | 19 and 6 |
| 12. Registration: | Registration number and registry name. |  |
