# Supplementary material for: A systematic review and meta-analysis of the serum lipid profile in prediction of diabetic neuropathy
Source: Sci Rep. 2021 Jan 12;11:499. doi: 10.1038/s41598-020-79276-0 (PMC7804465; doi:10.1038/s41598-020-79276-0)
Supplement: Supplementary file 2 — Supplementary Information 2. [file 41598_2020_79276_MOESM2_ESM.docx]

# Full electronic search

Full electronic search performed in multiple international databases.

- PubMed
- Embase
- Cochrane Library
- Web of Science

|  |
| --- |
|  |

### PubMed search formula

#1) “Search ((Diabetic Neuropathy) OR Neuropathies, Diabetic) OR Neuropathy, Diabetic

#2) “Search ((((((serum lipid profiles) OR lipid profiles) OR lipid levels) OR triglycerides) OR total cholesterol) OR high-density lipoprotein cholesterol) OR low-density lipoprotein cholesterol

#3) “Search ((Diabetic Neuropathy) OR Neuropathies, Diabetic) OR Neuropathy, Diabetic

AND ((((((serum lipid profiles) OR lipid profiles) OR lipid levels) OR triglycerides) OR total cholesterol) OR high-density lipoprotein cholesterol) OR low-density lipoprotein cholesterol

### Web of science search formula

Timespan = All Years(1950-2020), Search language to use = English

#1) TS = “Diabetic Neuropathy” OR TS = “Neuropathies, Diabetic” OR TS = “Neuropathy, Diabetic”

#2) TS = “serum lipid profiles”OR TS = “lipid profiles” OR TS = “lipid levels” OR TS = “triglycerides” OR TS = “total cholesterol” OR TS = “high-density lipoprotein cholesterol” OR TS = “low-density lipoprotein cholesterol”

#3) #2 AND #1

### Embase Search

#1) ‘Diabetic Neuropathy’/exp OR ‘Neuropathies, Diabetic’ OR ‘Neuropathy, Diabetic’

#2) ‘serum lipid profiles’/exp OR ‘lipid profiles’ OR ‘lipid levels’/exp OR ‘triglycerides’ OR ‘total cholesterol’ OR ‘high-density lipoprotein cholesterol’ OR ‘low-density lipoprotein cholesterol’/exp

#3) #1 AND #2

#4) #3 AND (‘clinical trial’/de OR ‘randomized controlled trial’/de)

#5) #3 AND (‘clinical trial’/exp OR ‘clinical trial’ OR ‘randomized controlled trial’/exp OR ‘randomized controlled trial’)

#6) #3 AND (‘clinical trial’/exp OR ‘clinical trial’ OR ‘randomized controlled trial’/exp OR ‘randomized controlled trial’) AND ([controlled clinical trial]/lim OR [randomized controlled trial]/lim)

#7) #3 AND (‘clinical trial’/exp OR ‘clinical trial’ OR ‘randomized controlled trial’/exp OR ‘randomized controlled trial’) AND ([controlled clinical trial]/lim OR [randomized controlled trial]/lim) AND [embase]/lim

Cochrane Library Search formula

#1) Diabetic Neuropathy or Neuropathies, Diabetic or Neuropathy, Diabetic

#2) serum lipid profiles or lipid profiles or lipid levels or triglycerides or total cholesterol or high-density lipoprotein cholesterol or low-density lipoprotein cholesterol

#3) #1 and #2 and #3

#4) MeSH descriptor: [Diabetic Neuropathy] explode all trees

#5) MeSH descriptor: [Neuropathies, Diabetic] explode all trees

#6) MeSH descriptor: [Neuropathy, Diabetic] explode all trees

#7) MeSH descriptor: [serum lipid profiles] explode all trees

#8) MeSH descriptor: [lipid profiles] explode all trees

#9) MeSH descriptor: [lipid levels] explode all trees

#10) MeSH descriptor: [triglycerides] explode all trees

#11) MeSH descriptor: [total cholesterol] explode all trees

#12) MeSH descriptor: [high-density lipoprotein cholesterol] explode all trees

#13) MeSH descriptor: [low-density lipoprotein cholesterol] explode all trees

#14 #4 or #5 or #6

#15) #7 or #8 or #9 or #10 or #11 or #12 or #13

#16) #14 and #15

#17) #16 or #3

 #18) #17 or #3 in Trials
