# Supplementary material for: A systematic review and meta-analysis of the serum lipid profile in prediction of diabetic neuropathy
Source: Sci Rep. 2021 Jan 12;11:499. doi: 10.1038/s41598-020-79276-0 (PMC7804465; doi:10.1038/s41598-020-79276-0)
Supplement: Supplementary file 3 — Supplementary Information 3. [file 41598_2020_79276_MOESM3_ESM.docx]

The full search terms and strategies of the present review have been

published on PROSPERO (Reference: CRD42018094241).

Appendix A: Search strategy

Database: PubMed, Embase, Cochrane Library and Web of Science databases up to May, 2020.

Strategy:

1. “Diabetic Neuropathy”
2. “Neuropathies, Diabetic”
3. “Neuropathy, Diabetic”
4. “Diabetic Autonomic Neuropathy”
5. “Autonomic Neuropathies, Diabetic”
6. “Autonomic Neuropathies, Diabetic”
7. “Diabetic Autonomic Neuropathies”
8. “Neuropathies, Diabetic Autonomic”
9. “Neuropathy, Diabetic Autonomic”
10. “Diabetic Neuralgia”
11. “Diabetic Neuralgias”
12. “Neuralgias, Diabetic”
13. “Diabetic Neuropathy, Painful”
14. “Diabetic Neuropathies, Painful”
15. “Neuropathies, Painful Diabetic”
16. “Neuropathy, Painful Diabetic”
17. “Painful Diabetic Neuropathies”
18. “Painful Diabetic Neuropathy”
19. “Neuralgia, Diabetic”
20. “Symmetric Diabetic Proximal Motor Neuropathy”
21. “Asymmetric Diabetic Proximal Motor Neuropathy”
22. “Diabetic Asymmetric Polyneuropathy”
23. “Asymmetric Polyneuropathies, Diabetic”
24. “Asymmetric Polyneuropathy, Diabetic”
25. “Diabetic Asymmetric Polyneuropathies”
26. “Polyneuropathies, Diabetic Asymmetric”
27. “Polyneuropathy, Diabetic Asymmetric”
28. “Diabetic Mononeuropathy”
29. “Diabetic Mononeuropathies”
30. “Mononeuropathies, Diabetic”
31. “Mononeuropathy, Diabetic”
32. “Diabetic Mononeuropathy Simplex”
33. “Diabetic Mononeuropathy Simplices”
34. “Mononeuropathy Simplex, Diabetic”
35. “Mononeuropathy Simplices, Diabetic”
36. “Simplex, Diabetic Mononeuropathy”
37. “Simplices, Diabetic Mononeuropathy”
38. “Diabetic Amyotrophy”
39. “Amyotrophies, Diabetic”
40. “Amyotrophy, Diabetic”
41. “Diabetic Amyotrophies”
42. “Diabetic Polyneuropathy”
43. “Diabetic Polyneuropathies”
44. “Polyneuropathies, Diabetic”
45. “Polyneuropathy, Diabetic”
46. “serum lipid profiles”
47. “lipid profiles”
48. “lipid levels”
49. “triglycerides”
50. “total cholesterol”
51. “high-density lipoprotein cholesterol”
52. “low-density lipoprotein cholesterol”
